# Supplementary material for: Perceptions of risk in pregnancy with chronic disease: A systematic review and thematic synthesis
Source: PLoS One. 2021 Jul 19;16(7):e0254956. doi: 10.1371/journal.pone.0254956 (PMC8289065; doi:10.1371/journal.pone.0254956)
Supplement: S1 Table — (DOCX) [file pone.0254956.s002.docx]

# S1 Table. Search strategies for each database

**Database: EMBASE**

| 1 | risk perception*.mp. |
| --- | --- |
| 2 | perception* of risk.mp. |
| 3 | self-rated risk.mp. |
| 4 | pregnan* risk.mp. |
| 5 | (risk adj2 perception*).mp. [mp=title, abstract, heading word, drug trade name, original title, device manufacturer, drug manufacturer, device trade name, keyword, floating subheading word, candidate term word] |
| 6 | risk appraisal*.mp. |
| 7 | "perceived risk".mp. |
| 8 | (apprais* adj2 risk).mp. [mp=title, abstract, heading word, drug trade name, original title, device manufacturer, drug manufacturer, device trade name, keyword, floating subheading word, candidate term word] |
| 9 | self reported risk.mp. |
| 10 | (self adj2 risk).mp. [mp=title, abstract, heading word, drug trade name, original title, device manufacturer, drug manufacturer, device trade name, keyword, floating subheading word, candidate term word] |
| 11 | 1 or 2 or 3 or 4 or 5 or 6 or 7 or 8 or 9 or 10 |
| 12 | chronic disease/pc [Prevention] |
| 13 | "chronic medical condition*".mp. |
| 14 | chronic.mp. |
| 15 | (illness* or disease* or condition*).mp. [mp=title, abstract, heading word, drug trade name, original title, device manufacturer, drug manufacturer, device trade name, keyword, floating subheading word, candidate term word] |
| 16 | 14 and 15 |
| 17 | kidney disease.mp. |
| 18 | diabet*.mp. |
| 19 | hypertensi*.mp. |
| 20 | chronic rheumatological disease.mp. |
| 21 | chronic lung disease.mp. |
| 22 | coronary heart disease.mp. |
| 23 | epilepsy.mp. |
| 24 | (chronic adj2 disease).mp. [mp=title, abstract, heading word, drug trade name, original title, device manufacturer, drug manufacturer, device trade name, keyword, floating subheading word, candidate term word] |
| 25 | high risk pregnan*.mp. |
| 26 | 12 or 13 or 16 or 17 or 18 or 19 or 20 or 21 or 22 or 23 or 24 or 25 |
| 27 | exp high risk pregnancy/ or high risk pregnan*.mp. |
| 28 | exp attitude to pregnancy/ |
| 29 | "pregnancy risk".mp. |
| 30 | pregnan*.mp. |
| 31 | pregnan* intention*.mp. |
| 32 | prenatal care.mp. |
| 33 | exp pre pregnancy care/ |
| 34 | 27 or 28 or 29 or 30 or 31 or 32 or 33 |
| 35 | 11 and 26 and 34 |

**Database: Global Health**

| 1 | risk perception*.mp. |
| --- | --- |
| 2 | perception* of risk.mp. |
| 3 | self-rated risk.mp. |
| 4 | pregnan* risk.mp. |
| 5 | (risk adj2 perception*).mp. [mp=abstract, title, original title, broad terms, heading words, identifiers, cabicodes] |
| 6 | risk appraisal*.mp. |
| 7 | "perceived risk".mp. |
| 8 | (apprais* adj2 risk).mp. |
| 9 | self reported risk.mp. |
| 10 | (self adj2 risk).mp. |
| 11 | 1 or 2 or 3 or 4 or 5 or 6 or 7 or 8 or 9 or 10 |
| 12 | chronic disease.mp. or chronic diseases/ |
| 13 | "chronic medical condition*".mp. |
| 14 | chronic.mp. |
| 15 | (illness* or disease* or condition*).mp. [mp=abstract, title, original title, broad terms, heading words, identifiers, cabicodes] |
| 16 | 14 and 15 |
| 17 | kidney disease.mp. |
| 18 | diabet*.mp. |
| 19 | hypertensi*.mp. |
| 20 | chronic rheumatological disease.mp. |
| 21 | chronic lung disease.mp. |
| 22 | coronary heart disease.mp. |
| 23 | epilepsy.mp. |
| 24 | (chronic adj2 disease).mp. [mp=abstract, title, original title, broad terms, heading words, identifiers, cabicodes] |
| 25 | high risk pregnan*.mp. |
| 26 | 12 or 13 or 16 or 17 or 18 or 19 or 20 or 21 or 22 or 23 or 24 or 25 |
| 27 | high risk pregnan*.mp. |
| 28 | "pregnancy risk".mp. |
| 29 | pregnan*.mp. |
| 30 | pregnant women/ |
| 31 | pregnancy complications/ |
| 32 | prenatal care.mp. |
| 33 | 27 or 28 or 29 or 30 or 31 or 32 |
| 34 | 11 and 26 and 33 |

**Database: Medline**

| 1 | risk perception*.mp. |
| --- | --- |
| 2 | perception* of risk.mp. |
| 3 | self-rated risk.mp. |
| 4 | pregnan* risk.mp. |
| 5 | (risk adj2 perception*).mp. [mp=title, abstract, original title, name of substance word, subject heading word, floating sub-heading word, keyword heading word, organism supplementary concept word, protocol supplementary concept word, rare disease supplementary concept word, unique identifier, synonyms] |
| 6 | (self adj2 risk).mp. [mp=title, abstract, original title, name of substance word, subject heading word, floating sub-heading word, keyword heading word, organism supplementary concept word, protocol supplementary concept word, rare disease supplementary concept word, unique identifier, synonyms] |
| 7 | risk appraisal*.mp. |
| 8 | "perceived risk".mp. |
| 9 | (apprais* adj2 risk).mp. [mp=title, abstract, original title, name of substance word, subject heading word, floating sub-heading word, keyword heading word, organism supplementary concept word, protocol supplementary concept word, rare disease supplementary concept word, unique identifier, synonyms] |
| 10 | self reported risk.mp. |
| 11 | Chronic Disease/pc [Prevention & Control] |
| 12 | "chronic medical condition*".mp. |
| 13 | chronic.mp. |
| 14 | (illness* or disease* or condition*).mp. [mp=title, abstract, original title, name of substance word, subject heading word, floating sub-heading word, keyword heading word, organism supplementary concept word, protocol supplementary concept word, rare disease supplementary concept word, unique identifier, synonyms] |
| 15 | 13 and 14 |
| 16 | kidney disease.mp. |
| 17 | diabet*.mp. |
| 18 | hypertensi*.mp. |
| 19 | coronary heart disease.mp. |
| 20 | epilepsy.mp. |
| 21 | (chronic adj2 disease).mp. [mp=title, abstract, original title, name of substance word, subject heading word, floating sub-heading word, keyword heading word, organism supplementary concept word, protocol supplementary concept word, rare disease supplementary concept word, unique identifier, synonyms] |
| 22 | high risk preg*.mp. |
| 23 | chronic rheumatological disease.mp. |
| 24 | chronic lung disease.mp. |
| 25 | 11 or 12 or 15 or 16 or 17 or 19 or 20 or 21 or 22 or 23 or 24 |
| 26 | high risk preg*.mp. |
| 27 | high risk.mp. |
| 28 | pregnan*.mp. |
| 29 | 27 and 28 |
| 30 | Pregnancy/ |
| 31 | pregnan* intent*.mp. |
| 32 | prenatal care.mp. |
| 33 | Pregnancy Complications/ |
| 34 | prepregnancy care.mp. |
| 35 | 26 or 29 or 30 or 31 or 32 or 33 or 34 |
| 36 | (risk adj2 percep*).mp. |
| 37 | 1 or 2 or 3 or 4 or 5 or 6 or 7 or 8 or 9 or 10 or 36 |
| 38 | 25 and 35 and 37 |

**Database: SCOPUS**

(({risk perception}) OR ("perception* of risk") OR ("self rated risk") OR ("pregnan* risk") OR (risk w/2 perception*) OR ({risk appraisal}) OR ({perceived risk}) OR#11 OR ({self reported risk}) OR (risk perception)) AND ((chronic AND disease* OR illness* OR condition*) OR ({chronic medical condition}) OR ({kidney disease}) OR {diabetes} OR {diabetic} OR {hypertension} OR ({chronic rheumatological disease}) OR ({chronic lung disease}) OR ({coronary heart disease}) OR ({epilepsy}) OR ("chronic W/2 disease") OR ({high risk pregnancy}) OR ("high risk pregnancy")) AND (("high risk pregnancy") OR ("pregnancy risk") OR ({pregnancy}) OR ({pregnant}) OR ({pregnancies}) OR ({prenatal care}) OR ("pre pregnancy counselling") OR ({high risk pregnancy}) OR ({pregnancy complications}))

**Database: PSYCH Info**

| 1 | exp Risk Perception/ or Risk perception*.mp. |
| --- | --- |
| 2 | perception* of risk.mp. |
| 3 | self-rated risk.mp. |
| 4 | (risk adj2 perception*).mp. [mp=title, abstract, heading word, table of contents, key concepts, original title, tests & measures] |
| 5 | risk appraisal*.mp. |
| 6 | "perceived risk".mp. |
| 7 | (apprais* adj2 risk).mp. [mp=title, abstract, heading word, table of contents, key concepts, original title, tests & measures] |
| 8 | self reported risk.mp. |
| 9 | (self adj2 risk).mp. [mp=title, abstract, heading word, table of contents, key concepts, original title, tests & measures] |
| 10 | pregnan* risk.mp. |
| 11 | 1 or 2 or 3 or 4 or 5 or 6 or 7 or 8 or 9 or 10 |
| 12 | chronic illness.mp. or exp Chronic Illness/ |
| 13 | "chronic medical condition*".mp. |
| 14 | chronic.mp. |
| 15 | (illness* or disease* or condition*).mp. [mp=title, abstract, heading word, table of contents, key concepts, original title, tests & measures] |
| 16 | 14 and 15 |
| 17 | kidney disease.mp. |
| 18 | diabet*.mp. |
| 19 | hypertensi*.mp. |
| 20 | chronic rheumatological disease.mp. |
| 21 | chronic lung disease.mp. |
| 22 | coronary heart disease.mp. |
| 23 | epilepsy.mp. |
| 24 | (chronic adj2 disease).mp. [mp=title, abstract, heading word, table of contents, key concepts, original title, tests & measures] |
| 25 | high risk.mp. |
| 26 | pregnan*.mp. |
| 27 | 25 and 26 |
| 28 | 12 or 13 or 16 or 17 or 18 or 19 or 20 or 21 or 22 or 23 or 24 or 27 |
| 29 | exp Pregnancy/ |
| 30 | "pregnancy risk".mp. |
| 31 | pregnan*.mp. |
| 32 | pregnan* intention*.mp. |
| 33 | prenatal care.mp. |
| 34 | prepregnancy counselling.mp. |
| 35 | 29 or 30 or 31 or 32 or 33 or 34 |
| 36 | 11 and 28 and 35 |

**Database: Cumulative Index of Nursing and Allied Health Literature**

| S36 | S12 AND S28 AND S35 |
| --- | --- |
| S35 | S29 OR S30 OR S31 OR S32 OR S33 OR S34 |
| S34 | "pre pregnancy counselling" |
| S33 | "prenatal care" |
| S32 | "pregnan* intention*" |
| S31 | (MH "Attitude to Pregnancy") |
| S30 | "pregnan*" |
| S29 | (MH "Pregnancy, High Risk") OR "high risk pregnancy" |
| S28 | S13 OR S14 OR S15 OR S16 OR S17 OR S18 OR S19 OR S20 OR S21 OR S22 OR S23 OR S24 OR S25 OR S26 OR S27 |
| S27 | "high risk pregnan*" |
| S26 | "high risk pregnancy" |
| S25 | ""chronic n2 illness"" |
| S24 | ""chronic n2 disease"" |
| S23 | "epilepsy" |
| S22 | "coronary heart disease" |
| S21 | "chronic lung disease" |
| S20 | "chronic rheumatological disease" |
| S19 | "hypertensi*" |
| S18 | "diabet*" |
| S17 | "kidney disease" |
| S16 | "chronic condition*" |
| S15 | ""chronic illness"" |
| S14 | ""chronic medical condition*"" |
| S13 | (MH "Chronic Disease+") OR "chronic disease" |
| S12 | S1 OR S2 OR S3 OR S4 OR S5 OR S6 OR S7 OR S8 OR S9 OR S10 OR S11 |
| S11 | "self n2 risk" |
| S10 | ""self reported risk"" |
| S9 | ""appraisal n2 risk"" |
| S8 | ""perceived risk"" |
| S7 | (MH "Attitude to Risk") |
| S6 | ""risk appraisal"" |
| S5 | ""risk N2 perception*"" |
| S4 | "pregnan* risk" |
| S3 | "self-rated risk" |
| S2 | "risk perception*" |
| S1 | (MH "Attitude to Risk") OR "risk perception" |

**Database: Web of Science**

| # 33 | #32 AND #26 AND #12 |
| --- | --- |
|  | Indexes=SCI-EXPANDED, SSCI, A&HCI, CPCI-S, CPCI-SSH, ESCI Timespan=All years |
| # 32 | #31 OR #30 OR #29 OR #28 OR #27 |
|  | Indexes=SCI-EXPANDED, SSCI, A&HCI, CPCI-S, CPCI-SSH, ESCI Timespan=All years |
| # 31 | TS= "pre pregnancy counselling" |
|  | Indexes=SCI-EXPANDED, SSCI, A&HCI, CPCI-S, CPCI-SSH, ESCI Timespan=All years |
| # 30 | TS= "prenatal care" |
|  | Indexes=SCI-EXPANDED, SSCI, A&HCI, CPCI-S, CPCI-SSH, ESCI Timespan=All years |
| # 29 | TS="pregnan* intention*" |
|  | Indexes=SCI-EXPANDED, SSCI, A&HCI, CPCI-S, CPCI-SSH, ESCI Timespan=All years |
| # 28 | TS="pregnan*" |
|  | Indexes=SCI-EXPANDED, SSCI, A&HCI, CPCI-S, CPCI-SSH, ESCI Timespan=All years |
| # 27 | TS="high risk pregnan*" |
|  | Indexes=SCI-EXPANDED, SSCI, A&HCI, CPCI-S, CPCI-SSH, ESCI Timespan=All years |
| # 26 | #25 OR #24 OR #23 OR #22 OR #21 OR #20 OR #19 OR #18 OR #17 OR #16 OR #15 OR #14 OR #13 |
|  | Indexes=SCI-EXPANDED, SSCI, A&HCI, CPCI-S, CPCI-SSH, ESCI Timespan=All years |
| # 25 | TS="high risk pregnan*" |
|  | Indexes=SCI-EXPANDED, SSCI, A&HCI, CPCI-S, CPCI-SSH, ESCI Timespan=All years |
| # 24 | TS= (chronic NEAR/2 disease*) |
|  | Indexes=SCI-EXPANDED, SSCI, A&HCI, CPCI-S, CPCI-SSH, ESCI Timespan=All years |
| # 23 | TS="epilepsy" |
|  | Indexes=SCI-EXPANDED, SSCI, A&HCI, CPCI-S, CPCI-SSH, ESCI Timespan=All years |
| # 22 | TS="coronary heart disease" |
|  | Indexes=SCI-EXPANDED, SSCI, A&HCI, CPCI-S, CPCI-SSH, ESCI Timespan=All years |
| # 21 | TS="chronic lung disease" |
|  | Indexes=SCI-EXPANDED, SSCI, A&HCI, CPCI-S, CPCI-SSH, ESCI Timespan=All years |
| # 20 | TS="chronic rheumatological disease" |
|  | Indexes=SCI-EXPANDED, SSCI, A&HCI, CPCI-S, CPCI-SSH, ESCI Timespan=All years |
| # 19 | TS="hypertension" |
|  | Indexes=SCI-EXPANDED, SSCI, A&HCI, CPCI-S, CPCI-SSH, ESCI Timespan=All years |
| # 18 | TS="diabetes" |
|  | Indexes=SCI-EXPANDED, SSCI, A&HCI, CPCI-S, CPCI-SSH, ESCI Timespan=All years |
| # 17 | TS="kidney disease" |
|  | Indexes=SCI-EXPANDED, SSCI, A&HCI, CPCI-S, CPCI-SSH, ESCI Timespan=All years |
| # 16 | TS="chronic medical condition*" |
|  | Indexes=SCI-EXPANDED, SSCI, A&HCI, CPCI-S, CPCI-SSH, ESCI Timespan=All years |
| # 15 | TS= "Chronic condition*" |
|  | Indexes=SCI-EXPANDED, SSCI, A&HCI, CPCI-S, CPCI-SSH, ESCI Timespan=All years |
| # 14 | TS="Chronic illness*" |
|  | Indexes=SCI-EXPANDED, SSCI, A&HCI, CPCI-S, CPCI-SSH, ESCI Timespan=All years |
| # 13 | TS= (chronic disease*) |
|  | Indexes=SCI-EXPANDED, SSCI, A&HCI, CPCI-S, CPCI-SSH, ESCI Timespan=All years |
| # 12 | #10 OR #9 OR #8 OR #7 OR #6 OR #5 OR #4 OR #3 OR #2 OR #1 |
|  | Indexes=SCI-EXPANDED, SSCI, A&HCI, CPCI-S, CPCI-SSH, ESCI Timespan=All years |
| # 11 | TS= ("perceive AND risk") |
|  | Indexes=SCI-EXPANDED, SSCI, A&HCI, CPCI-S, CPCI-SSH, ESCI Timespan=All years |
| # 10 | TS=(self NEAR/2 risk) |
|  | Indexes=SCI-EXPANDED, SSCI, A&HCI, CPCI-S, CPCI-SSH, ESCI Timespan=All years |
| # 9 | TS= "Self reported risk" |
|  | Indexes=SCI-EXPANDED, SSCI, A&HCI, CPCI-S, CPCI-SSH, ESCI Timespan=All years |
| # 8 | TS=(Appraisal* NEAR/2 risk) |
|  | Indexes=SCI-EXPANDED, SSCI, A&HCI, CPCI-S, CPCI-SSH, ESCI Timespan=All years |
| # 7 | TS= "risk appraisal" |
|  | Indexes=SCI-EXPANDED, SSCI, A&HCI, CPCI-S, CPCI-SSH, ESCI Timespan=All years |
| # 6 | TS="percept* of risk" |
|  | Indexes=SCI-EXPANDED, SSCI, A&HCI, CPCI-S, CPCI-SSH, ESCI Timespan=All years |
| # 5 | TS= (risk NEAR/2 perception*) |
|  | Indexes=SCI-EXPANDED, SSCI, A&HCI, CPCI-S, CPCI-SSH, ESCI Timespan=All years |
| # 4 | TS= "pregnan* risk" |
|  | Indexes=SCI-EXPANDED, SSCI, A&HCI, CPCI-S, CPCI-SSH, ESCI Timespan=All years |
| # 3 | TS="self-rated risk" |
|  | Indexes=SCI-EXPANDED, SSCI, A&HCI, CPCI-S, CPCI-SSH, ESCI Timespan=All years |
| # 2 | TS= "perception* of risk" |
|  | Indexes=SCI-EXPANDED, SSCI, A&HCI, CPCI-S, CPCI-SSH, ESCI Timespan=All years |
| # 1 | TS = (risk perception*) |
|  | Indexes=SCI-EXPANDED, SSCI, A&HCI, CPCI-S, CPCI-SSH, ESCI Timespan=All years |
