# Supplementary material for: Perceptions of risk in pregnancy with chronic disease: A systematic review and thematic synthesis
Source: PLoS One. 2021 Jul 19;16(7):e0254956. doi: 10.1371/journal.pone.0254956 (PMC8289065; doi:10.1371/journal.pone.0254956)
Supplement: S2 Table — (DOCX) [file pone.0254956.s003.docx]

# S2 Table. Quality assessment of included qualitative papers using the Critical Appraisal Skills Programme Qualitative checklist (2018)

| **Paper** | **Re-viewer** | | **1. Was there a clear statement of the aims of the research?** | **2.Is a qualitative methodology appropriate?** | **3. Was the research design appropriate to address the aims of the research?** | **4. Was the recruitment strategy appropriate to the aims of the research?** | **5. Was the data collected in a way that addressed the research issue?** | **6. Has the relationship between researcher and participants been adequately considered?** | **7. Have ethical issues been taken into consideration?** | **8. Was the data analysis sufficiently rigorous?** | **9. Is there a clear statement of findings?** | **10. Is the research valuable?*** |
| --- | --- | --- | --- | --- | --- | --- | --- | --- | --- | --- | --- | --- |
| Rodrigues et al (2020) | | PS | Yes | Yes | Yes | Yes | Yes | Yes | Yes | Yes | Yes | Yes |
|  |  | ER | Yes | Yes | Yes | Yes | Yes | No | Yes | Yes | Yes | Yes |
| Mccorry et al (2012) | | PS | Yes | Yes | Yes | Yes | Yes | Yes | Yes | Yes | Yes | Yes |
|  |  | ER | Yes | Yes | Yes | Yes | Yes | Yes | Yes | Yes | Yes | Yes |
| Widnes, Schjøtt, Granas (2012) | | PS | Yes | Yes | Yes | Yes | Yes | Can’t Tell | Yes | Yes | Yes | Yes |
|  |  | ER | Yes | Yes | Yes | Yes | Yes | Yes | Yes | Yes | Yes | Yes |
| Boardman (2013) | | PS | Yes | Yes | Yes | Yes | Yes | Yes | Yes | Yes | Yes | Yes |
|  |  | ER | Yes | Yes | Yes | Yes | Yes | Yes | Yes | Yes | Yes | Yes |
| Tyer-viola and Lopez (2014) | | PS | Yes | Yes | Yes | Yes | Yes | Yes | Yes | Yes | Yes | Yes |
|  |  | ER | Yes | Yes | Yes | Yes | Yes | No | Yes | Yes | Yes | Yes |
| Ngu, Hay and Menahem (2014) | | PS | Yes | Yes | Yes | Can’t Tell | Yes | Can’t Tell | Yes | No | Yes | Yes |
|  |  | ER | Yes | Yes | Can’t Tell | Can’t Tell | Yes | Can’t Tell | Yes | No | Yes | Yes |
| Wotherspoon et al (2017) | | PS | Yes | Yes | Yes | Yes | Yes | Yes | Yes | Yes | Yes | Yes |
|  |  | ER | Yes | Yes | Yes | Yes | Yes | Yes | Yes | Yes | Yes | Yes |
| Singh et al | | PS | Yes | Yes | Yes | Yes | Yes | No | Yes | Yes | Yes | Yes |
|  |  | ER | Yes | Yes | Yes | Yes | Yes | No | Yes | Yes | Yes | Yes |
| *N/B. Item 10 was adapted from a free text response “How valuable is the research” | | | | | | | | | | | | |
