# Supplementary material for: Perceptions of risk in pregnancy with chronic disease: A systematic review and thematic synthesis
Source: PLoS One. 2021 Jul 19;16(7):e0254956. doi: 10.1371/journal.pone.0254956 (PMC8289065; doi:10.1371/journal.pone.0254956)
Supplement: S1 Record — (PDF) [file pone.0254956.s004.pdf]

National Institute for Health Research

UNIVERSITY of York
Centre for Reviews and Dissemination

Systematic review

1. * Review title.

Give the title of the review in English

Perceptions of risk in pregnancy with chronic disease: A systematic review and thematic synthesis

2. Original language title.

For reviews in languages other than English, give the title in the original language. This will be displayed with the English language title.

3. * Anticipated or actual start date.

Give the date the systematic review started or is expected to start.

15/04/2019

4. * Anticipated completion date.

Give the date by which the review is expected to be completed.

01/04/2020

5. * Stage of review at time of this submission.

Tick the boxes to show which review tasks have been started and which have been completed. Update this field each time any amendments are made to a published record.

Reviews that have started data extraction (at the time of initial submission) are not eligible for inclusion in PROSPERO. If there is later evidence that incorrect status and/or completion date has been supplied, the published PROSPERO record will be marked as retracted.

This field uses answers to initial screening questions. It cannot be edited until after registration.

The review has not yet started: No

Review stage	Started	Completed
Preliminary searches	Yes	Yes
Piloting of the study selection process	Yes	Yes
Formal screening of search results against eligibility criteria	Yes	Yes
Data extraction	Yes	Yes
Risk of bias (quality) assessment	Yes	Yes
Data analysis	Yes	Yes

Provide any other relevant information about the stage of the review here.

The review has been completed and is being submitted for publication.

The review has been completed and is being submitted for publication.

6. * Named contact.

The named contact is the guarantor for the accuracy of the information in the register record. This may be any member of the review team.

Elizabeth R Ralston

Email salutation (e.g. "Dr Smith" or "Joanne") for correspondence:

Miss Ralston

7. * Named contact email.

Give the electronic email address of the named contact.

elizabeth.ralston@kcl.ac.uk

8. Named contact address

Give the full institutional/organisational postal address for the named contact.

3rd Floor, Addison House, Kings College London, SE1 1UL

9. Named contact phone number.

Give the telephone number for the named contact, including international dialling code.

10. * Organisational affiliation of the review.

Full title of the organisational affiliations for this review and website address if available. This field may be completed as 'None' if the review is not affiliated to any organisation.

King's College London

Organisation web address:

https://www.kcl.ac.uk/

11. * Review team members and their organisational affiliations.

Give the personal details and the organisational affiliations of each member of the review team. Affiliation refers to groups or organisations to which review team members belong. **NOTE: email and country now MUST be entered for each person, unless you are amending a published record.**

Miss Elizabeth Ralston. King's College London
Dr Kate Bramham. King's College London
Dr Priscilla Smith. Guy's and St Thomas' NHS Foundation Trust
Dr Joseph Chilcot. King's College London
Mr Sergio Silverio. King's College London

12. * Funding sources/sponsors.

Details of the individuals, organizations, groups, companies or other legal entities who have funded or sponsored the review.

Studentship funding to Elizabeth Ralston from King’s Medical Research Trust, Joint Research Committee

Grant number(s)

State the funder, grant or award number and the date of award

13. * Conflicts of interest.

List actual or perceived conflicts of interest (financial or academic).

None

14. Collaborators.

Give the name and affiliation of any individuals or organisations who are working on the review but who are not listed as review team members. **NOTE: email and country must be completed for each person, unless you are amending a published record.**

15. * Review question.

State the review question(s) clearly and precisely. It may be appropriate to break very broad questions down into a series of related more specific questions. Questions may be framed or refined using PI(E)COS or similar where relevant.

The objective is to systematically review the qualitative literature to describe risk perceptions of pregnancy in women with chronic diseases.

16. * Searches.

State the sources that will be searched (e.g. Medline). Give the search dates, and any restrictions (e.g. language or publication date). Do NOT enter the full search strategy (it may be provided as a link or attachment below.)

The following electronic databases will be used to identify relevant research.

There are no time restrictions for the data extraction. The time frames for each database are highlighted in corresponding brackets.

o MEDLINE (1946 to 2020)

o Scopus (1996 to 2020)

o PsycINFO (1960 to 2020)

o EMBASE (1974 to 2020)

o Global Health (1973 to 2020)

o Web of Science (1900 to 2020)

o CINAHL (Cumulative Index to Nursing and Allied Health Literature – 1981 to 2020)

To reduce publication bias, grey literature will also be searched. Grey literature will be searched using the following sources:

o OpenGrey

o British Library Electronic Theses Online Service (EThOS)

o Dart European (Digital Access to Research Theses Europe)

o OpenAIRE

17. URL to search strategy.

Upload a file with your search strategy, or an example of a search strategy for a specific database, (including the keywords) in pdf or word format. In doing so you are consenting to the file being made publicly accessible. Or provide a URL or link to the strategy. Do NOT provide links to your search **results**.

https://www.crd.york.ac.uk/PROSPEROFILES/132367_STRATEGY_20190415.pdf

Alternatively, upload your search strategy to CRD in pdf format. Please note that by doing so you are consenting to the file being made publicly accessible.

Do not make this file publicly available until the review is complete

18. * Condition or domain being studied.

Give a short description of the disease, condition or healthcare domain being studied in your systematic review.

Risk perception is an individual's subjective appraisal of potential harm (Brewer et al., 2007). This subjective appraisal of risk is composed of three dimensions; the perceived severity of the potential harm, the perceived likelihood of the harm occurring and the perceived susceptibility of the individual to the harm (Brewer et al., 2007; Darker 2013). This review is exploring women's risk perceptions in pregnancy with chronic disease.

19. * Participants/population.

Specify the participants or populations being studied in the review. The preferred format includes details of both inclusion and exclusion criteria.

Women who are currently pregnant (not restrictive to gestational age), experienced pregnancy or planning a pregnancy (including those attending pre-pregnancy counselling), who have a diagnosis of a chronic medical condition or long-term condition prior to pregnancy. This includes; chronic kidney disease, chronic lung disease, chronic rheumatological disease, diabetes, obesity, hypertension, epilepsy, coronary heart disease.

20. * Intervention(s), exposure(s).

Give full and clear descriptions or definitions of the interventions or the exposures to be reviewed. The preferred format includes details of both inclusion and exclusion criteria.

Not applicable.

21. * Comparator(s)/control.

Where relevant, give details of the alternatives against which the intervention/exposure will be compared (e.g. another intervention or a non-exposed control group). The preferred format includes details of both inclusion and exclusion criteria.

Not applicable.

22. * Types of study to be included.

Give details of the study designs (e.g. RCT) that are eligible for inclusion in the review. The preferred format includes both inclusion and exclusion criteria. If there are no restrictions on the types of study, this should be stated.

Qualitative study designs are eligible for the review. Quantitative (including; cross-sectional, cohort, randomised control trials, pre/post intervention, observational, case-control), meta-analysis and systematic reviews will be excluded. There are no time restrictions on publication.

Papers must be published in English and fully accessible. If only the abstract can be reviewed it will be excluded. Previously restricting to published in English would have introduced a language bias, although as English is now regarded as the universal language of science (Morrisson et al., 2012), the extent of introducing a language bias is reduced (Higgins and Green, 2011).

23. Context.

Give summary details of the setting or other relevant characteristics, which help define the inclusion or exclusion criteria.

24. * Main outcome(s).

Give the pre-specified main (most important) outcomes of the review, including details of how the outcome is defined and measured and when these measurement are made, if these are part of the review inclusion criteria.

The primary outcome of this review is describing women's risk perceptions in pregnancy with chronic conditions. Data items regarding women's risk perceptions will be extracted. Qualitative responses, including women's risk appraisal and perceptions of maternal and neonatal risk.

Measures of effect

Please specify the effect measure(s) for you main outcome(s) e.g. relative risks, odds ratios, risk difference, and/or 'number needed to treat.

Not applicable.

25. * Additional outcome(s).

List the pre-specified additional outcomes of the review, with a similar level of detail to that required for main outcomes. Where there are no additional outcomes please state 'None' or 'Not applicable' as appropriate to the review

None.

Measures of effect

Please specify the effect measure(s) for you additional outcome(s) e.g. relative risks, odds ratios, risk difference, and/or 'number needed to treat.

Not applicable.

26. * Data extraction (selection and coding).

Describe how studies will be selected for inclusion. State what data will be extracted or obtained. State how this will be done and recorded.

The selection process will be guided by PRISMA statement. The data will initially be identified from the search and extracted, with duplicates removed. Titles and abstracts will be screened for eligibility. Two independent reviewers (ER & PS) will assess the full texts with the inclusion and exclusion criteria. The discrepancies will be discussed, if no consensus is agreed a third individual will be consulted.

27. * Risk of bias (quality) assessment.

State which characteristics of the studies will be assessed and/or any formal risk of bias/quality assessment tools that will be used.

The papers included in the final review will be assessed for within study-risk of bias. Critical Appraisal Skills Program Qualitative List will be used to assess the qualitative research. Two researchers (ER and PS) will independently assess the quality of the publications.

28. * Strategy for data synthesis.

Describe the methods you plan to use to synthesise data. This **must not be generic text** but should be **specific to your review** and describe how the proposed approach will be applied to your data. If meta-analysis is planned, describe the models to be used, methods to explore statistical heterogeneity, and software package to be used.

A thematic synthesis will be provided with the information presented in the text and tables to summarise and explain the findings and characteristics of the included studies. The narrative synthesis will follow guidance from Thomas and Harden (2008).

29. * Analysis of subgroups or subsets.

State any planned investigation of ‘subgroups’. Be clear and specific about which type of study or participant will be included in each group or covariate investigated. State the planned analytic approach.

There are no plans for analysis of subgroups.

30. * Type and method of review.

Select the type of review, review method and health area from the lists below.

Type of review

Cost effectiveness
No

Diagnostic
No

Epidemiologic
No

Individual patient data (IPD) meta-analysis
No

Intervention
No

Meta-analysis
No

Methodology
No

Narrative synthesis
Yes

Network meta-analysis
No

Pre-clinical
No

Prevention
No

Prognostic
No

Prospective meta-analysis (PMA)
No

Review of reviews
No

Service delivery
No

Synthesis of qualitative studies
Yes

Systematic review
Yes

Other
No

Health area of the review

Alcohol/substance misuse/abuse
No

Blood and immune system
No

Cancer
No

Cardiovascular
No

Care of the elderly
No

Child health
No

Complementary therapies
No

COVID-19
No

Crime and justice
No

Dental
No

Digestive system
No

Ear, nose and throat

No

Education
No

Endocrine and metabolic disorders
No

Eye disorders
No

General interest
No

Genetics
No

Health inequalities/health equity
No

Infections and infestations
No

International development
No

Mental health and behavioural conditions
No

Musculoskeletal
No

Neurological
No

Nursing
No

Obstetrics and gynaecology
No

Oral health
No

Palliative care
No

Perioperative care
No

Physiotherapy
No

Pregnancy and childbirth
Yes

Public health (including social determinants of health)
No

Rehabilitation
No

Respiratory disorders
No

Service delivery
No

Skin disorders
No

Social care
No

Surgery
No

Tropical Medicine
No

Urological
No

Wounds, injuries and accidents
No

Violence and abuse
No

31. Language.

Select each language individually to add it to the list below, use the bin icon to remove any added in error.
English

There is an English language summary.

32. * Country.

Select the country in which the review is being carried out. For multi-national collaborations select all the countries involved.

England

33. Other registration details.

Name any other organisation where the systematic review title or protocol is registered (e.g. Campbell, or The Joanna Briggs Institute) together with any unique identification number assigned by them. If extracted data will be stored and made available through a repository such as the Systematic Review Data Repository (SRDR), details and a link should be included here. If none, leave blank.

34. Reference and/or URL for published protocol.

If the protocol for this review is published provide details (authors, title and journal details, preferably in Vancouver format)

Add web link to the published protocol.

Or, upload your published protocol here in pdf format. Note that the upload will be publicly accessible.

No I do not make this file publicly available until the review is complete

Please note that the information required in the PROSPERO registration form must be completed in full even if access to a protocol is given.

35. Dissemination plans.

Do you intend to publish the review on completion?

Yes

Give brief details of plans for communicating review findings.?

The systematic review is planned to be published on completion. As well as the abstract being submitted for a poster presentation at a conference within the United Kingdom.

36. Keywords.

Give words or phrases that best describe the review. Separate keywords with a semicolon or new line. Keywords help PROSPERO users find your review (keywords do not appear in the public record but are included in searches). Be as specific and precise as possible. Avoid acronyms and abbreviations unless these are in wide use.

Risk perception; pregnancy; chronic illness; health beliefs

37. Details of any existing review of the same topic by the same authors.

If you are registering an update of an existing review give details of the earlier versions and include a full bibliographic reference, if available.

38. * Current review status.

Update review status when the review is completed and when it is published.New registrations must be ongoing so this field is not editable for initial submission.
Please provide anticipated publication date

Review_Completed_not_published

39. Any additional information.

Provide any other information relevant to the registration of this review.

40. Details of final report/publication(s) or preprints if available.

Leave empty until publication details are available OR you have a link to a preprint (NOTE: this field is not editable for initial submission). List authors, title and journal details preferably in Vancouver format.

Give the link to the published review or preprint.
